# Identification of *Fusarium virguliforme* FvTox1-Interacting Synthetic Peptides for Enhancing Foliar Sudden Death Syndrome Resistance in Soybean

**DOI:** 10.1371/journal.pone.0145156

**Published:** 2015-12-28

**Authors:** Bing Wang, Sivakumar Swaminathan, Madan K. Bhattacharyya

**Affiliations:** Department of Agronomy, Iowa State University, Ames, 50011–1010, United States of America; The Chinese University of Hong Kong, HONG KONG

## Abstract

Soybean is one of the most important crops grown across the globe. In the United States, approximately 15% of the soybean yield is suppressed due to various pathogen and pests attack. Sudden death syndrome (SDS) is an emerging fungal disease caused by *Fusarium virguliforme*. Although growing SDS resistant soybean cultivars has been the main method of controlling this disease, SDS resistance is partial and controlled by a large number of quantitative trait loci (QTL). A proteinacious toxin, FvTox1, produced by the pathogen, causes foliar SDS. Earlier, we demonstrated that expression of an anti-FvTox1 single chain variable fragment antibody resulted in reduced foliar SDS development in transgenic soybean plants. Here, we investigated if synthetic FvTox1-interacting peptides, displayed on M13 phage particles, can be identified for enhancing foliar SDS resistance in soybean. We screened three phage-display peptide libraries and discovered four classes of M13 phage clones displaying FvTox1-interacting peptides. *In vitro* pull-down assays and *in vivo* interaction assays in yeast were conducted to confirm the interaction of FvTox1 with these four synthetic peptides and their fusion-combinations. One of these peptides was able to partially neutralize the toxic effect of FvTox1 *in vitro*. Possible application of the synthetic peptides in engineering SDS resistance soybean cultivars is discussed.

## Introduction

Sudden death syndrome (SDS) is an emerging disease caused by the fungal pathogen, *Fusarium virguliforme*. Between 1999 and 2004, the average annual yield suppression due to SDS was estimated to be $190 million [1]. The disease was first recorded in Arkansas in 1971 [2]. Now the pathogen has been detected in all soybean-growing states of North America [3]. The disease has two components: (i) foliar SDS and (ii) root necrosis. Major crop losses occur from the foliar SDS. *F*. *virguliforme* is a soil borne pathogen. It over-winters in crop residues or soil in the form of chlamydospores that initiate root-infection in subsequent years. The pathogen has never been detected in the above ground diseased tissues. The application of fungicides in furrow during planting or as seed treatments has little success in controlling this fungal pathogen; and similarly, foliar application of fungicides has little success on controlling the disease because the foliar symptoms are caused by toxins produced by the pathogen in infected roots [4–8].

The *F*. *virguliforme* can be maintained in culture media. Earlier, a 17 kDa protein was purified from the *F*. *virguliforme* culture filtrate that causes necrosis on detached wounded soybean cotyledons [5]. The pathogen releases a large number of proteins to the culture medium [6]. One of these proteins, FvTox1, has been shown to cause foliar SDS [7]. Investigation of knockout *fvtox1* mutants established that FvTox1 is the major toxin for foliar SDS development in soybean [8]. The toxin requires light to cause foliar SDS symptoms [7,9]. Expression of an anti-FvTox1 single-chain variable fragment antibody reduced foliar SDS development in transgenic soybean plants [10].

Growing of SDS resistant soybean cultivars has been the main method of controlling this disease. Unfortunately, the SDS resistance is partial and encoded by a large number of quantitative trait loci (QTL), each conditioning a small effect. Thus, breeding SDS resistant soybean cultivars is very challenging. Creation and application of alternative SDS resistance mechanisms is becoming urgent to complement the partial SDS resistance of soybean cultivars. As the foliar SDS is the most important component of the disease, generation of an anti-FvTox1 antibody to neutralize the toxicity of FvTox1 could improve foliar SDS resistance by complementing the partial resistance of soybean cultivars. Unfortunately, the anti-FvTox1 plant antibody designed earlier to enhance foliar SDS resistance in transgenic soybean plants [10] was developed based on mRNAs, extracted from a mammalian hybrid cell line; and therefore, soybeans of such transgenic plants are unsuitable for human consumption.

Like single variable fragment plant antibodies created based on mammalian mRNA molecules, linear peptides also have the ability to specifically bind and alter functions of target proteins. Compared to macromolecular antibodies, interacting peptides possess several attractive features. For example, they bear high structural compatibility and recognition specificity to the target proteins. Furthermore, small sizes allow peptides to cross cell membranes into intracellular compartments [11]. High structural compatibility and small sizes, make peptides more attractive to alter functions of target proteins [11,12]. *In vivo* or *in vitro* studies have shown that peptides can block functions of proteins including toxins and inhibit microbial infections [13–17]. A peptide with antibacterial activity has been identified from a phage display library [18]. Peptides can also be used as molecular diagnostic tools based on their binding affinity to certain target proteins or molecules [19–21].

Phage display is an extremely powerful strategy for isolating synthetic peptides that specifically bind to target proteins. In this technology, a library of synthetic oligonucleotides are fused to a coat protein gene so that a library of recombinant fusion peptides are displayed on the surface of the engineered bacteriophage for *in vitro* interaction with the target proteins. For example, in bacteriophage M13 displayed peptides are N-terminal fusions to the minor coat protein pIII that is involved in adhesion to bacterial F pilus for infection [22].

Over 50 peptide-based products, generated through phage display systems, have been approved for clinical uses [11]. There are a few examples of peptide-discovery to inhibit or monitor plant pathogens including virus, bacteria, fungi and nematode [19]. *Phakopsora pachyrhizi* causes Asian soybean rust, a devastating disease in many soybean-growing countries including Brazil and Argentina. Peptides isolated from a phage display library were able to inhibit growth of *P*. *pachyrhizi* germ tube when mixed with germinating spores [14]. For enhancing resistance of potatoes to nematodes, chemoreception disruptive peptide has been expressed in transgenic plants. These peptides have shown to suppress nematode parasitism up to 61% as compared to the non-transgenic control [23]. *In vitro* peptides binding to zoospores of the fungal pathogen *Phytophthora capsici* caused premature encystment of the zoospore [24].

One of the drawbacks of the peptides discovered using phage display technology is that peptides often bind to their targets with low affinities [16]. In animals, peptides are attached to a synthetic scaffold to enhance the potency of peptide-binding [25]. In an earlier study, peptides that bound to *P*. *capsici* zoospores were fused to maize cytokinine oxidase/dehydrogenase as a display scaffold. When peptides were secreted from transgenic tomato roots as fusion proteins with the maize cytokinin oxidase/dehydrogenase to the rhizosphere, they were effective in inducing premature zoospore encystment deterring zoospores from landing on the root surfaces resulting in enhanced root resistance to zoospore mediated infection [26].

Here we investigated if small, linear synthetic FvTox1-interacting peptides with ability to neutralize toxicity of FvTox1 can be identified from M13 phase display libraries for enhancing foliar SDS resistance in soybean. Our study revealed that one FvTox1-interacting peptide was able to partially suppress the toxicity effect of FvTox1 *in vitro*.

## Materials and Methods

### Phage Display Peptide Library Screening

Three phage display peptides libraries, Ph.D.-7, Ph.D.-12 and Ph.D-C7C were obtained from NEB Inc. (New England Lab, Woburn, MA). FvTox1 was expressed in an insect cell line and immobilized on a plate for screening the phage display libraries using a modified NEB, Inc. screening protocol ([7]; S1 File).

### Western Blotting

We developed an assay based on western blotting to identify the FvTox1 (His-tagged at C-terminus)-interacting phage particles, which is described in details in S1 File.

### Expression and Purification of His-Tagged Proteins in *E*. *coli*

Single strand DNA sequences of four isolated peptides along with nucleotides encoding GGGSGGGS linker were synthesized in Integrated DNA Technologies^®^ (Coralville, IA). For constructing peptide fusion genes, the PCR products of desired single peptide carrying complementary cohesive ends were ligated into expression vector pRSET (Life Technologies, Carlsbad, CA). Constructed plasmids were sequenced to avoid any mutations. To clone each synthetic gene into protein expression vector pET41, we designed two primers carrying either *Bam*HI or *Xho*I restriction site (S1 Table). For protein expression, constructed plasmids were transformed into *E*. *coli* BL21 (DE3) pLysS cells. When the *E*. *coli* BL21(DE3) pLysS cell lines were at OD600 0.6, the transformed plasmids were induced with 1 mM IPTG at room temperature for overnight. We purified soluble His-tagged proteins using Ni-NTA agarose (Qiagen, Valencia, CA).

Individual purified protein samples were filtered through Amicon Ultra-0.5 Centrifugal Filters for 3 kDa pore size (EMD Millipore, Billerica, MA). 10 units of thrombin was added to the recombinant protein, which was then filtered through a Amicon Ultra-0.5 Centrifugal Filter Units with 30 kDa pore size to obtain GST-tag free proteins. Purified protein samples were separated on SDS PAGE gels to determine their extent of purity. Protein concentrations were quantified using protein assay dye reagent concentrate (Bio-Rad Laboratories, Inc., Hercules, CA).

### Pull Down Assay

Pull down assay was conducted as suggested earlier [27] with some modifications, and is presented in details in S1 File.

### Yeast Two Hybrid and β-Galactosidase Activity Assay

Synthetic FvTox1-interacting peptides genes were PCR amplified and cloned into the pB42D vector. FvTox1 was cloned into the pLexA vector. FvTox1-pLexA plasmid was co-transformed with each synthetic FvTox1-interacting peptide gene in pB42AD into the yeast EGY48 [pSH18-34] isolate, which carries two reporters, LacZ and LEU2. The transformed cells were plated on minimal agar plates (SD/-His/-Trp/-Ura) to select colonies containing both plasmids. To test the activation of both reporter genes (LacZ and LEU2), 5 clones from each transformation were selected to individually inoculate 3 ml of SD/Glucose/-His/-Trp/-Ura liquid media and grew for overnight. Details of screening protocol and analyses of clones are presented in S1 File.

### Stem Cutting Assay

The stem cutting assay was conducted according to standard procedure [7]. Details of stem cut assays and analyses of the treated plants are presented in S1 File)

## Results

### Identification of M13 Phage Clones Displaying Putative FvTox1-Interacting Peptides

The target FvTox1 was expressed and purified from an insect line by following a protocol described earlier [7] and stored at -20°C (S1A Fig). The stem-cutting assay was performed to confirm that the purified FvTox1 was functional and can cause the typical interveinal chlorosis symptom. The leaves of soybean cultivar, ‘Williams 82’ fed with FvTox1 showed the typical interveinal chlorosis (S1B Fig).

**Fig 1:**
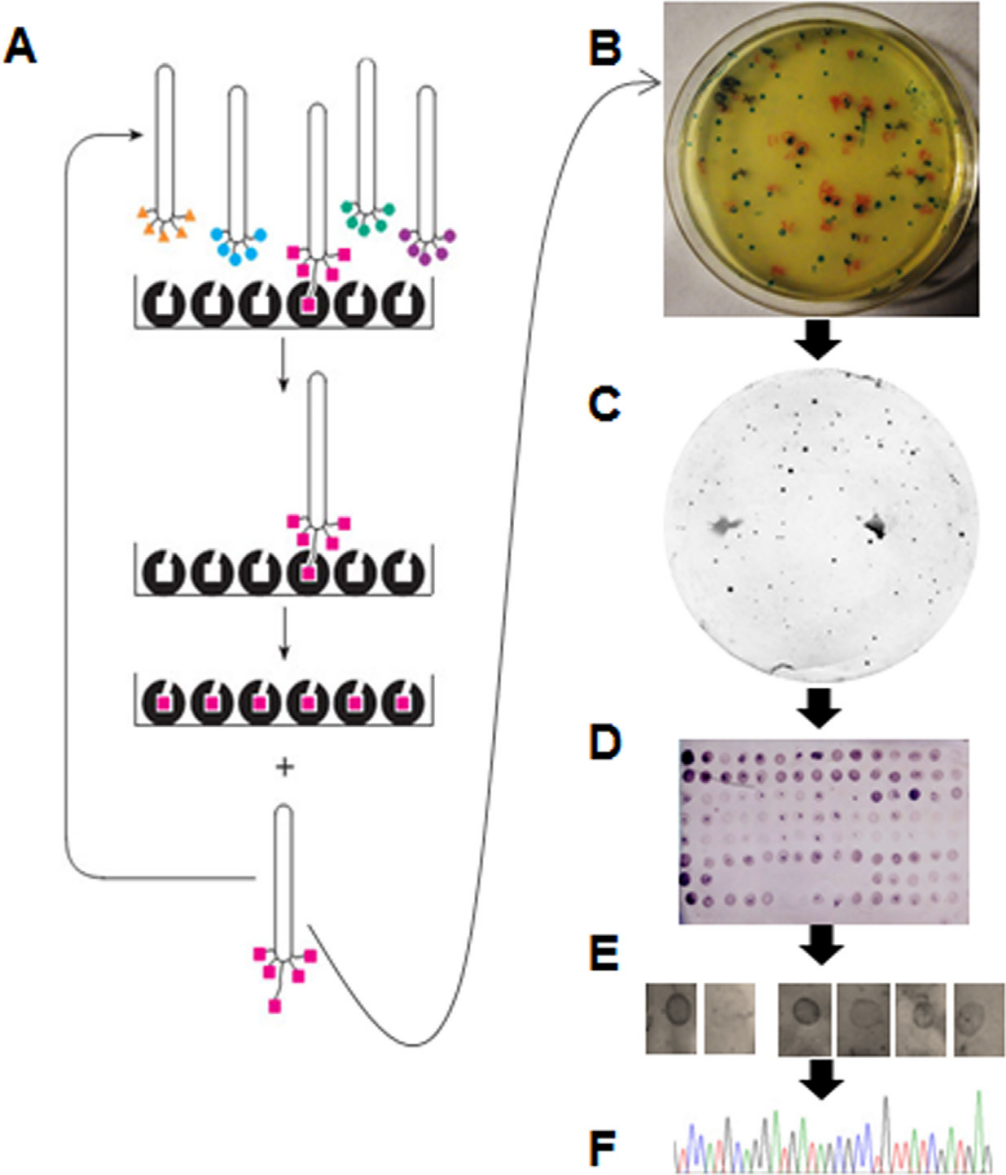
Diagrammatic representation of the workflow applied in affinity purification of M13 phage clones that displayed FvTox1-interacting synthetic peptides. (A), Bio-panning of the phage display libraries on plastic surface of a microtiter plates coated with 1.5 ml FvTox1 (30 ng/μl). Unbound phage particles were washed off; and M13 phage particles bound to FvTox1 were used to infect *E*. *coli* for starting a second round of panning. The process was repeated once more. (B), Plating of candidate M13 phage clones displaying FvTox1-interacting peptides. An eluate from the last panning in A was plated on X-gal/IPTG agar plates. (C), Identification of candidate M13 phage clones displaying FvTox1-interacting peptides. Phage clones were adsorbed onto nitrocellulose paper and hybridized to the His-tagged purified FvTox1 proteins. FvTox1-interacting clones were identified by detecting FvTox1 with the anti-His antibody. (D), Western blot analysis of the selected phage clones for interaction with FvTox1. Selected M13 phage particles from plates in C were transferred to nitrocellulose filters and hybridized to FvTox1, which was detected with an anti-His antibody. (E), Western blot analysis of the selected clones for interaction with FvTox1. Selected clones in D were reinvestigated for interaction with FvTox1, adsorbed onto a nitrocellulose membranes and detecting the interaction of individual clones to FvTox1 with an anti-M13 antibody (Details are presented in S2 Fig). (F), Electropherogram of a nucleotide molecule encoding an FvTox1-intearcting peptide is presented.

Three M13 phage display peptide libraries were mixed in equal proportions for panning using FvTox1, immobilized on 12-well microtiter plate surface (Fig 1A). In order to improve the stringency of panning, the amount of FvTox1 coated to the plate wells in the second and third rounds was reduced significantly (Table 1). At the same time the binding time was reduced from 60 to 30 min and the concentration of Tween-20 in washing buffer was increased from 0.1% to 0.5% (Table 1). The number of eluted phage particles in the third round of panning were increased 1,000 times compared to that in the second round of panning suggesting enrichment in FvTox1-interacting M13 phage particles.

**Table 1:** Bio-panning conditions for screening three M13 phage display libraries.

Panning Round	Input Phage (pfu/ml)	Recovered Phage (pfu/ml)	Binding Time (min)	Elution Time (min)	Tween-20 (%)	FvTox1 Concentration (ng/μl)^1^
**1**	2 × 10 ^11^	1.7 × 10^5^	60	20	0.1	30
**2**	10^11^	2.1 × 10^3^	30	20	0.5	1
**3**	10^11^	2 × 10^6^	30	20	0.5	1

^1^The volume of FvTox1 solution was 1.5 ml.

We conducted western blot analysis to identify the M13 phage clones displaying putative FvTox1-interacting peptides. Over 160 M13 clones were identified in the first round of western blotting. Selected clones were amplified by infecting *E*. *coli* ER2738 and plated onto LB agar amended with X-gal/IPTG for the second round of western blotting. Thirty-nine M13 positive clones were chosen from the second round of western blotting for sequencing (Fig 1C–1F).

### Classification of the Putative FvTox1-Interacting Phage-Displayed Peptides

Based on sequences of displayed peptides, we classified the 35 M13 phase clones identified through western blotting (Fig 1) into four classes. Class I contains 26 M13 phage clones, Class II seven phage clones, and Classes III and IV carry single phage clone each (Table 2).

**Table 2:** Four classes of the phage displayed peptides that interact with FvTox1.

Peptide	Phage Library	Phage clone	Sequence	pI/Da
**PEP1**	Ph.D. -12	1, 2, 4, 5, 6, 7, 8, 10, 11, 12, 13, 14, 15, 16, 17, 18, 19, 20, 21, 23, 28, 29, 30, 36, 38, 39	SYLPETIYEYRL	4.53/1,547
**PEP2**	Ph.D. -12	3, 9, 22, 25, 26, 27, 37	VENKTRYHDREV	6.73/1,546
**PEP3**	Ph.D. -12	24	HEGAWHNYARSV	6.92/1,427
**PEP4**	Ph.D. -7	31	SNGRVAD	5.55/718

### Generation and Expression of Synthetic FvTox1-Interacting Genes

**Fig 2:**
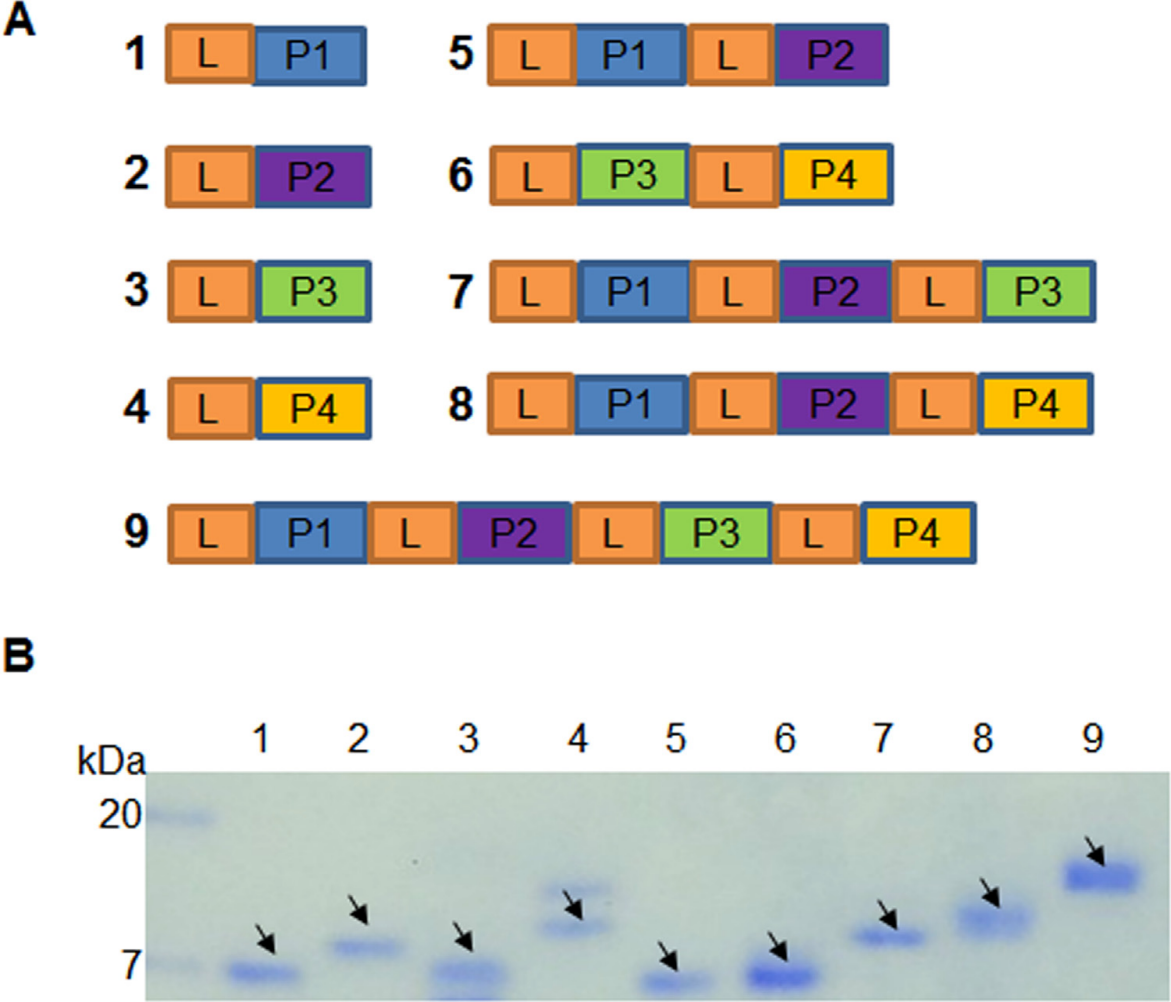
Expression of putative FvTox1-interacting peptides in *Escherchia coli*. (A), Schematic representation of nine fusion synthetic genes developed from four putative FvTox1-interacting peptide encoding genes isolated from the recombinant M13 phages (S2 Table). L represents linker. P1, P2, P3, and P4 are four peptides, PEP1, PEP2, PEP3, and PEP4, respectively, identified from four classes of phages, Classes 1, II, III and IV, respectively (Table 2). L, linker sequence GGGSGGGSGGGS. (B), Purified nine putative FvTox1-interacting proteins expressed from the nine synthetic genes (A) in *E*. *coli* (S2 Table). Arrows show the respective proteins.

In order improve the interaction of four classes of putative FvTox1-interacting peptides (Table 2) to FvTox1, we applied a PCR-based cloning approach to generate nine distinct fusion genes (Fig 2A; Table 3). DNA sequence encoding GGGS linkers were added to DNA sequences encoding the four classes of peptide (Table 2; S2 Table). At the initial stage, we used the pRSET expression vector, which carries the His and Xpress tags. Expression of five fusion peptides containing two or more of the four M13 displayed peptides was successful in this plasmid vector. We used pET41 plasmid vector carrying the GST tag to express the four M13 displayed peptides (single peptides). The GST tag was removed from the recombinant proteins through thrombin digestion (Fig 2B; S3 Table).

**Table 3:** Deduced amino acid sequences of nine synthetic peptide genes.

**Gene**	**Deduced amino acid sequence**
**^1^1**	GGGGSGGGGSGGGGSSYLPETIYEYRLGGGGS
**2**	GGGGSGGGGSGGGGSVENKTRYHDREVGGGGS
**3**	GGGGSGGGGSGGGGSHEGAWHNYARSVGGGGS
**4**	GGGGSGGGGSGGGGSSNGRVADGGGGS
**5**	GGGGSGGGGSGGGGSSYLPETIYEYRLGGGGSELGGGGSGGGGSGGGGSVENKTRYHDREVGGGGS
**6**	GGGSGGGGSGGGGSHEGAWHNYARSVGGGGSELGGGGSGGGGSGGGGSSNGRVADGGGGS
**7**	GSGGGGSGGGGSGGGGSSYLPETIYEYRLGGGGSELGGGGSGGGGSGGGGSVENKTRYHDREVGGGGSLEGGGGSGGGGSGGGGSHEGAWHNYARSVGGGGS
**8**	GGGGSGGGGSGGGGSSYLPETIYEYRLGGGGSELGGGGSGGGGSGGGGSVENKTRYHDREVGGGGSLEGGGGSGGGGSGGGGSSNGRVADGGGGS
**9**	GGGGSGGGGSGGGGSSYLPETIYEYRLGGGGSELGGGGSGGGGSGGGGSVENKTRYHDREVGGGGSLEGGGGSGGGGSGGGGSHEGAWHNYARSVGGGGSGTGGGGSGGGGSGGGGSSNGRVADGGGGS

^1^Displayed peptides (Table 2) used in creating the genes are underlined. Linkers are added to stich the displayed peptides in creating the synthetic genes.

### Interaction of Nine Fusion Peptides with FvTox1

To determine the strength of nine fusion peptides with FvTox1 (Fig 2), we applied two approaches: (i) *in vitro* pull-down assay, and (ii) *in vivo* interaction in yeast.

**Fig 3:**
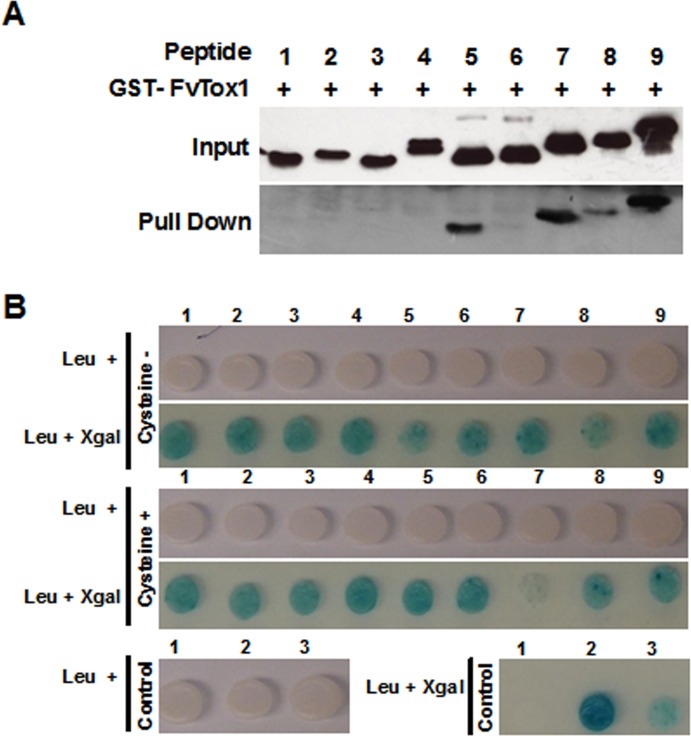
*In vitro* and *in vivo* interactions of putative FvTox1-interacting peptides with FvTox1. (A), Pull down assays of nine putative FvTox1-interacting peptides was conducted by binding the *E*. *coli* expressed fusion peptides (Fig 2) to FvTox1, which was immobilized on the GST-column. The FvTox1-interacting peptides pulled down by FvTox1 were detected with an anti-His antibody. The strengths of interactions between individual synthetic peptides with FvTox1 are presented in Table 4. (B), *In vivo* interactions of nine putative FvTox1-interacting fusion peptides with FvTox1 in a yeast two-hybrid system. Nine synthetic genes shown in Fig 3A, were cloned as fusion genes with the DNA activation domain of the pB42D plasmid. In nine additional constructs, two cysteine residues were added, one on each side the nine FvTox1-interacting peptides. β-galactosidase activities showing the extent of interaction of individual Fv-Tox1-interacting peptides with FvTox1 are presented in Table 4. Control 1, empty pB42AD vector. Control 2, soybean *GmTRX3* gene encoding a thioredoxin protein that interacts with FvTox1 (B. Wang and M.K. Bhattacharyya, unpublished). Control 3, soybean *GmGD1* gene encoding a glycine cleavage protein that interacts with FvTox1 (B. Wang and M.K. Bhattacharyya, unpublished).

In *in vitro* pull down assays, similar amounts of purified His-tagged peptide/fusion proteins were mixed with GST-tagged FvTox1 and pulled down the protein complex using glutathione resin. Western blot analysis of the pulled down protein complexes with the anti-His antibody revealed that fusion proteins, generated by fusing individual FvTox1-interacting phage displayed peptides, showed improved interaction with FvTox1 as compared to that of the individual single peptides with FvTox1 (Fig 3A; Table 4).

**Table 4:** *In vitro* and *in vivo* affinities of nine synthetic FvTox1-interacting peptides with FvTox1.

Peptide	^1^Pull down protein	^2^β-galactosidase activity (Miller units)	
		- cysteine	+ cysteine
1	3.9 ± 0.3	6.59 ± 1.17	6.31 ± 1.17
2	5.5 ± 1.8	5.88 ± 0.83	5.70 ± 1.09
3	2.7 ± 0.4	5.87 ± 1.07	6.74 ± 0.93
4	2.3 ± 0.7	6.45 ± 1.27	6.74 ± 1.66
5	31.8 ± 5.0	3.11 ± 0.53	5.62 ± 0.78
6	8.8 ± 5.4	5.32 ± 1.49	5.92 ± 1.48
7	38.5 ± 1.5	6.12 ± 1.23	1.21 ± 0.54
8	26.0 ± 7.8	3.35 ± 0.84	6.05 ± 1.30
9	47.0 ± 8.6	5.64 ± 1.56	5.81 ± 1.24
*GmTRX3*	-	9.64 ± 1.44	
*GmGD1*	-	5.79 ± 3.37	
Empty vector	0.00	0.48 ± .48	

^1,^ The data are from two independent experiments and show relative binding strength calculated in percent FvTox1-interacting peptides pulled down by FvTox1. The unit is not true reflection of percentages because the pulled down western blot was overexposed.

^2,^ Standard errors for β-galactosidase activity were calculated from data collected in three biological replications.

*In vitro* interaction provides only an indication of possible *in vivo* interactions between two proteins. To gain a better insight into the possible *in planta* interaction of the FvTox1-interacting peptides with FvTox1, we conducted *in vivo* interaction studies in yeast. Yeast two-hybrid assays were conducted using the LexA two-hybrid system. FvTox1 was expressed in the pLexA vector as a fusion to activation domain of the prokaryotic LexA transcription factor, while the nine peptides (Pep1 through Pep9; S4 Table) fused individually to the activation domain in the pB42AD plasmid. The interaction of FvTox1 to each of the nine peptides was observed in yeast (Fig 3B). However, quantitative β-galactocidase activity assays indicated that the five fusion proteins generated from four phage-displayed peptides were not better than the individual progenitor single peptides for interaction with FvTox1 (Table 4). Addition of a cysteine residue on each side of the individual peptides to improve structures of the Fv-Tox1 interacting peptides also did not improve any interaction strength of the nine peptides with FvTox1 (Table 4).

### Biological Activity of the Putative FvTox1-Interacting M13 Phage Displayed Peptides

**Fig 4:**
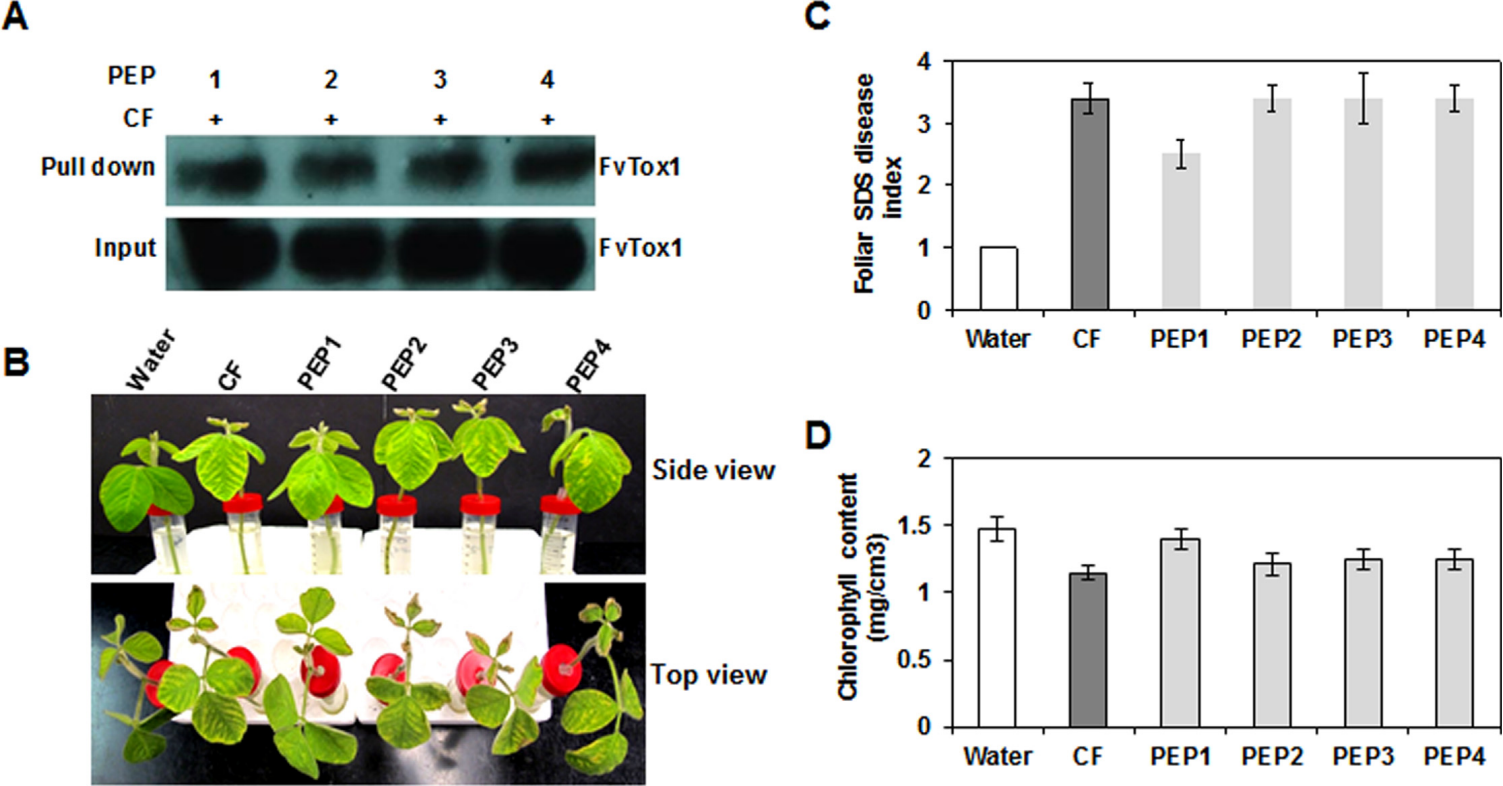
Reduced foliar SDS symptom development by cell-free *Fv* culture filtrates, pre-adsorbed with the FvTox1-interacting PEP1. (A), Chlorotic and necrotic leaf symptoms were recorded on day 8 following feeding of cut soybean seedlings with cell-free *Fv* culture filtrates that were pre-adsorbed with individual M13 phage displayed peptides (Table 5). (B), Reduced foliar SDS symptoms were induced in seedlings that were fed with cell-free *Fv* culture filtrates pre-adsorbed with PEP1 as compared to cell-free *Fv* culture filtrates (CF), or CF, pre-adsorbed with any of the other three peptides, PEP2, PEP3 or PEP4. (C), Reduced chlorophyll contents in all treatments except water control and CF pre-adsorbed with PEP1. (D), *In vitro* pull down assays of FvTox1 from CF using His-tagged FvTox1-interacting peptides (Table 5). FvTox1 was detected using anti-FvTox1 antibody [7]. Error bars indicate the standard errors calculated from means of three biological replications.

To investigate if any of the four single peptides identified through screening of phage display libraries can suppress foliar SDS symptom development, eight peptides with and without a His-tag added at the C-terminus of each putative FvTox1-interacting single peptide (PEP1 through PEP4; Table 5) were commercially synthesized (NeoBioLab, Woburn, MA). Considering the high cost of synthesizing longer peptides, we restricted our study to the four basic peptides. Individual single peptides were preincubated with the *F*. *virguliforme* culture filtrate, which causes foliar SDS in cut soybean seedlings [28]. Pre-incubation of PEP 1 with cell-free *F*. *virguliforme* culture filtrates significantly reduced the foliar SDS symptom development as compared to that following feeding cut soybean seedlings with only the cell-free *F*. *virguliforme* culture filtrates (Fig 4B and 4C, S3 Fig).

**Table 5:** Synthetic peptides used in determining FvTox1-neutralizing affect of displayed peptides *in vitro*.

Peptide	Deduced amino acid sequence
PEP1	SYLPETIYEYRL
PEP2	VENKTGYHDREV
PEP3	HEGAWHNYARSV
PEP4	SNGRVAD
PEP1-HIS	SYLPETIYEYRLHHHHH
PEP2-HIS	VENKTRYHDREVHHHHH
PEP3-HIS	HEGAWHNYARSVHHHHH
PEP4-HIS	SNGRVADHHHHH

## Discussion

Currently, there is no suitable soybean cultivar that is completely resistant to SDS. Expression of plant antibodies designed based on genetic information from mammals against pathogen proteins has been shown to protect plants from invading pathogens [29]. We have demonstrated earlier that expression of an anti-FvTox1 single-chain variable fragment antibody neutralizes the toxic effect of FvTox1 and enhances foliar SDS resistance [10]. Although our study established that expression of plant antibody against pathogen toxins could be a suitable strategy in enhancing resistance against toxin-induced plant diseases, the mouse-based anti-FvTox1 antibody expressed in soybean is not suitable for human consumption. We therefore investigated if artificial genes encoding FvTox1-interacting peptides can be created to neutralize FvTox1 for enhancing foliar SDS resistance in transgenic soybean lines.

Peptides can interact very specifically with proteins [30]. In plants, peptides have been shown to be useful in inhibiting pathogen infection or adhesion to host [14,21,23,26]. Phage display peptide-screening is an ideal approach to identify peptides that bind to a target protein. We have screened three M13 phage display peptide libraries (New England Lab, Woburn, MA) and discovered four classes of M13 phage clones encoding FvTox1-interacting peptides. Three classes of clones carry peptides of 12 amino acid (aa) residues; only one class of a single clone carries a peptide of seven aa residues. This may suggest that long peptides rather than the short peptides have the advantage of binding to the target, FvTox1. Of the 33 positive M13 clones sequenced, 25 carry the Class I peptide suggesting that PEP1 probably strongly interact with FvTox1 as compared to the other three peptides under the conditions of library screening. Alternatively, it could also be due to decreased viability or growth of M13 clones carrying PEP2, PEP3 and PEP4 as compared to those carrying PEP1 (Table 2). Expression of some displayed peptides as fusion proteins with M13 pIII protein involved in adhesion to bacterial F pilus required for host infection could decrease the infectivity of M13 phage particles [22].

The four FvTox1-interacting peptides did not show any conserved residues suggesting that the peptides may interact to different epitopes of FvTox1 and fusing the peptides could improve the binding affinity to FvTox1, which was apparent from pull down assays of at least few peptides generated by fusing two or more of the phage-displayed peptides (Fig 3A; Table 4).

We applied multiple approaches to confirm the binding affinity of the peptides to FvTox1. Both *in vitro* and *in vivo* protein-protein interaction studies established that the isolated four peptides should be suitable to determine their possible role in neutralizing FvTox1 toxin for enhancing foliar SDS in transgenic soybean plants. *In vitro* binding of FvTox1 of cell-free *F*. *virguliforme* culture filtrates with each of the four phage-displayed peptides indicated that at least one peptide (PEP1) was able to neutralize the toxic effect of FvTox1 and reduce foliar SDS development to some extent in stem cutting assays as compared to the control (Fig 4).

We added two cysteine residues, one on each side of the nine synthetic peptides, for improving the binding affinities of the nine fusion peptides to FvTox1 (Fig 3). It is expected that formation of the sulfhydryl bridges between the added two flanking cys residues could improve the binding affinities of the FvTox1-interacting peptides to FvTox1. When two flanking cys residues were added, the *in vivo* binding affinity of FvTox1 with two (Pep5 and Pep8) of the five fusion peptides in yeast was improved as compared to their corresponding original forms with no cys residues at their flanking sites (Table 4). On the contrary, reduced interaction strength of FvTox1 with PEP7 with two flanking cys residues was observed as compared to that of FvTox1 with PEP7 with no cys residues (Fig 3; Table 4). These results suggest that expression of one or more of the 18 FvTox1-interacting peptides could neutralize FvTox1 *in planta* and enhance foliar SDS resistance in transgenic soybean plants.

In mammals, antibodies bind to target using six antibody complementarity determining regions [31]. The single peptides have weaker affinity compared to antibody. In animals, to increase affinity of binding single peptides to target proteins many copies of peptides are attached to synthetic scaffold such as liposome [16,25]. In plants, peptides can be fused to selected proteins as display scaffold for delivering to correct cellular or extracellular spaces [26]. FvTox1 is the major toxin that induces foliar SDS. The toxin protein has been localized to chloroplasts (H.K. Brar and M.K. Bhattacharyya, unpublished). Light is essential for FvTox1-induced foliar SDS symptoms [7,9]. A thioredoxin protein, GmTRX2, localized to chloroplasts, has been identified as the candidate FvTox1-interacting target soybean protein (R.N. Pudake, and M.K. Bhattacharyya, unpublished). Targeting the FvTox1-interacting peptides to chloroplasts using a suitable chloroplast protein such as the FvTox1-interacting GmTRX2 as display scaffold could compete with endogenous FvTox1-interacting protein for FvTox1 binding and thereby suppress the foliar SDS development in transgenic soybean plants. If successful, this could be a suitable biotechnological approach for enhancing SDS resistance in soybean.

## Supporting Information

S1 Fig Purified FvTox1 produced typical chlorotic and necrotic foliar SDS symptoms in soybean. (A), Electrophoresis of purified His-tagged FvTox1 protein expressed in an Sf21 insect cell line. FvTox1 separated on a 12% SDS-PAGE gel was visualized by silver staining. (B), Interveinal chlorosis and necrosis of leaves developed in cut soybean Williams 82 seedlings fed with either cell-free *F*. *virguliforme* Mont-1 culture filtrate (Mont-1) or purified FvTox1 protein (FvTox1). Water, water control.
(PPTX)

S2 Fig Interaction of M13 phage clones with FvTox1. A drop of 3 μl of FvTox1 (100 ng/μl) was placed on each strip of nitrocellulose membrane buffer and air-dried. C1, a membrane was hybridized to M13 phage particles (1×10^14^ pfu) in PBS buffer. After overnight incubation of the strip with M13 phage particles at 4°C, strip was hybridized to the primary anti-M13 monoclonal antibody, and then to anti-mouse secondary antibody. C2, strip was first hybridized to anti-FvTox1 monoclonal antibody [7] and then to a secondary anti-mouse antibody (New England Lab, Woburn, MA). P1, M13 phage (#29) containing Pep1; P2, M13 phage (#26) containing Pep2; P3, M13 phage (24) containing Pep3; P4, M13 phage (#31) containing Pep4 (Table 2). For hybridization of FvTox1 with M13 phage particles, each strip was immersed in an individual tube containing a selected phage clone to a final concentration of 1×10^14^ pfu in PBS buffer. After overnight incubation of the strips with individual phage particles at 4°C, strips were hybridized to the primary anti-M13 monoclonal antibody and subsequently with to a secondary anti-mouse secondary antibody.
(PPTX)

S3 Fig Foliar SDS symptom development by cell-free *Fv* culture filtrates preincubated with the FvTox1-interacting peptides. Chlorotic and necrotic leaf symptoms were recorded on day 8 following feeding of cut soybean seedlings with cell-free *Fv* culture filtrates that were pre-adsorbed with individual M13 phage displayed peptides with no His tags (Table 5).
(PPTX)

S1 File Materials and Methods. (DOCX)

S1 Table Primers used in this study. (DOCX)

S2 Table Nucleotide sequences of all nine peptides. (DOCX)

S3 Table Fusion peptides expressed in *E*. *coli* for pull down assay. (DOCX)

S4 Table Plasmid constructs included in this study. (DOCX)
