# Supplementary figures and images for: Identification of Fusarium virguliforme FvTox1-Interacting Synthetic Peptides for Enhancing Foliar Sudden Death Syndrome Resistance in Soybean
Source: PLoS One. 2015 Dec 28;10(12):e0145156. doi: 10.1371/journal.pone.0145156 (PMC4692527; doi:10.1371/journal.pone.0145156)

## Slide 1
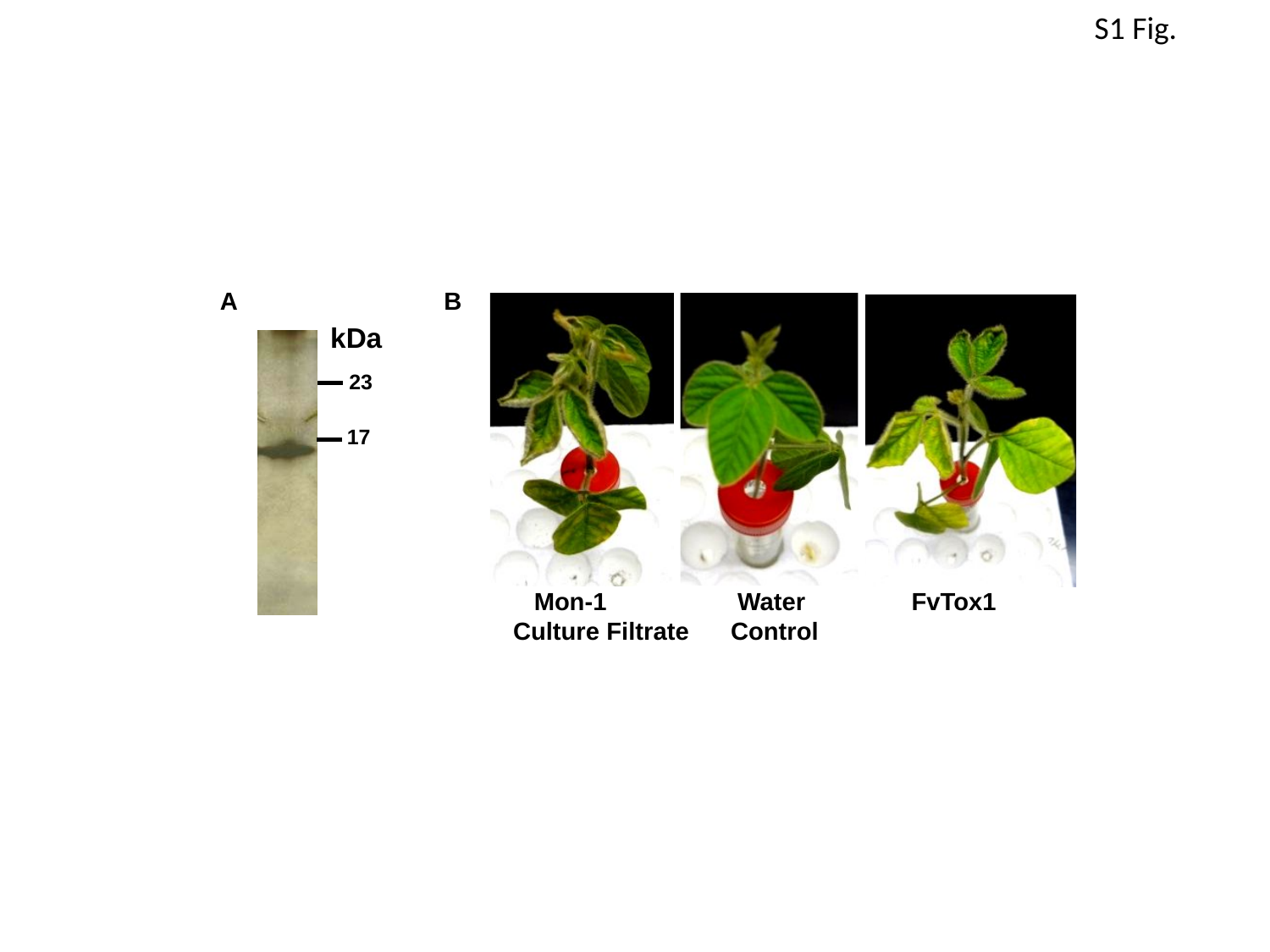

S1 Fig.
A
B
kDa
23
17
 Mon-1
Culture Filtrate
FvTox1
 Water Control

Supplement: S1 Fig — (A), Electrophoresis of purified His-tagged FvTox1 protein expressed in an Sf21 insect cell line. FvTox1 separated on a 12% SDS-PAGE gel was visualized by silver staining. (B), Interveinal chlorosis and necrosis of leaves developed in cut soybean Williams 82 seedlings fed with either cell-free F. virguliforme Mont-1 culture filtrate (Mont-1) or purified FvTox1 protein (FvTox1). Water, water control. (PPTX) [file pone.0145156.s001.pptx]

## Slide 1
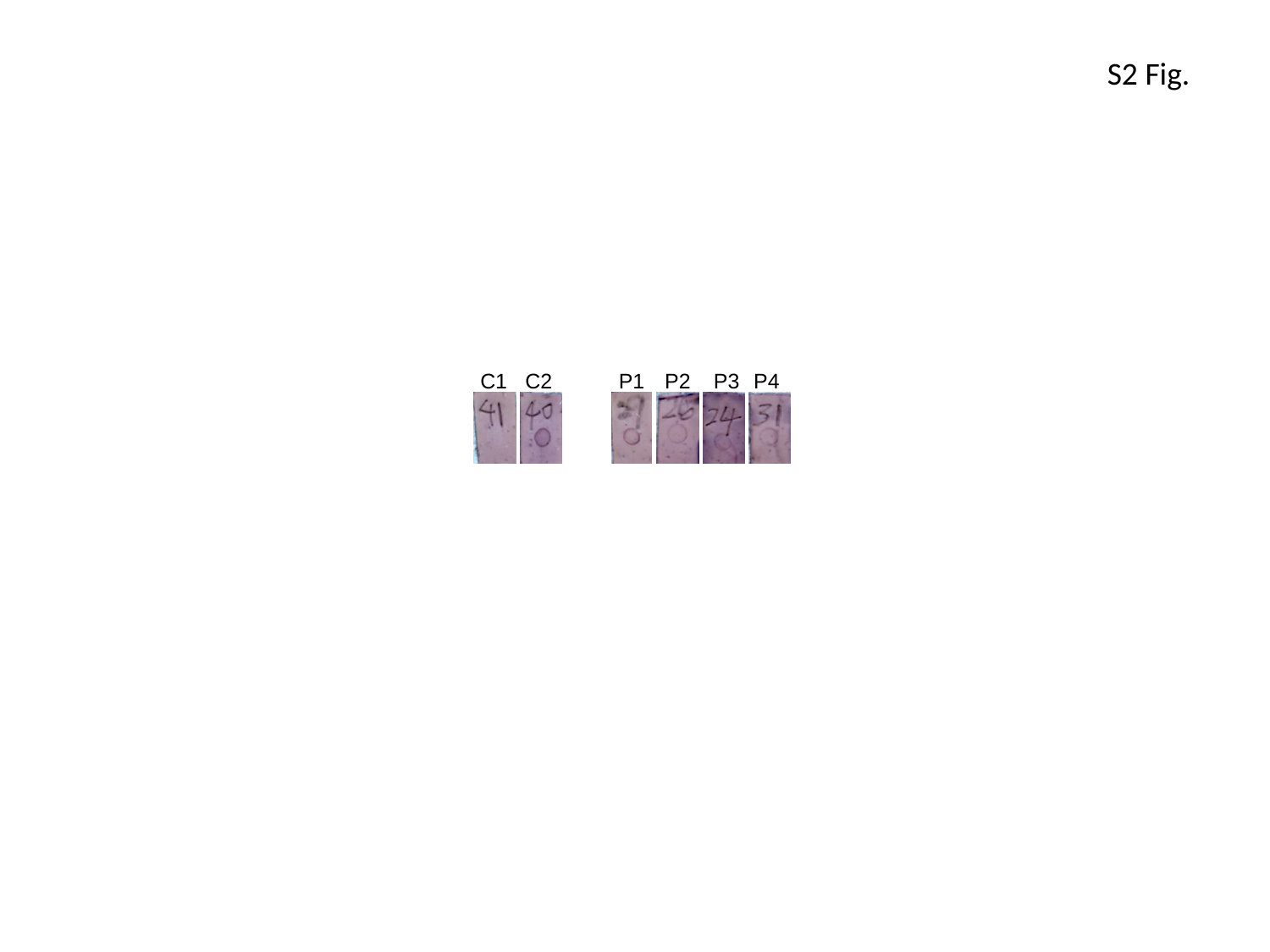

S2 Fig.
C1
C2
P1
P2
P3
P4

Supplement: S2 Fig — A drop of 3 μl of FvTox1 (100 ng/μl) was placed on each strip of nitrocellulose membrane buffer and air-dried. C1, a membrane was hybridized to M13 phage particles (1×10^14^ pfu) in PBS buffer. After overnight incubation of the strip with M13 phage particles at 4°C, strip was hybridized to the primary anti-M13 monoclonal antibody, and then to anti-mouse secondary antibody. C2, strip was first hybridized to anti-FvTox1 monoclonal antibody [7] and then to a secondary anti-mouse antibody (New England Lab, Woburn, MA). P1, M13 phage (#29) containing Pep1; P2, M13 phage (#26) containing Pep2; P3, M13 phage (24) containing Pep3; P4, M13 phage (#31) containing Pep4 (Table 2). For hybridization of FvTox1 with M13 phage particles, each strip was immersed in an individual tube containing a selected phage clone to a final concentration of 1×10^14^ pfu in PBS buffer. After overnight incubation of the strips with individual phage particles at 4°C, strips were hybridized to the primary anti-M13 monoclonal antibody and subsequently with to a secondary anti-mouse secondary antibody. (PPTX) [file pone.0145156.s002.pptx]

## Slide 1
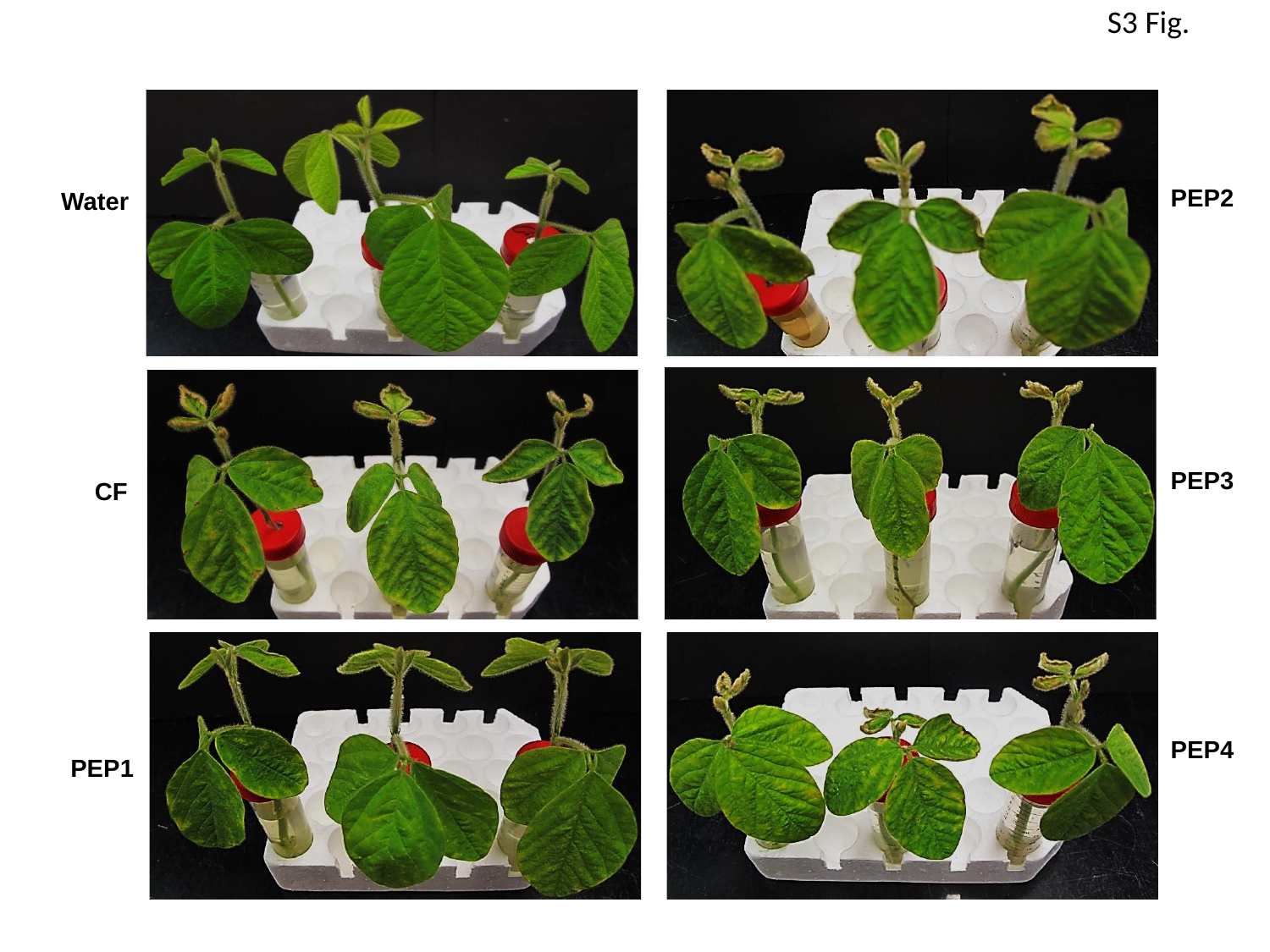

S3 Fig.
PEP2
Water
PEP3
CF
PEP4
PEP1

Supplement: S3 Fig — Chlorotic and necrotic leaf symptoms were recorded on day 8 following feeding of cut soybean seedlings with cell-free Fv culture filtrates that were pre-adsorbed with individual M13 phage displayed peptides with no His tags (Table 5). (PPTX) [file pone.0145156.s003.pptx]
