# Supplementary material for: Identification of Fusarium virguliforme FvTox1-Interacting Synthetic Peptides for Enhancing Foliar Sudden Death Syndrome Resistance in Soybean
Source: PLoS One. 2015 Dec 28;10(12):e0145156. doi: 10.1371/journal.pone.0145156 (PMC4692527; doi:10.1371/journal.pone.0145156)
Supplement: S1 File — (DOCX) [file pone.0145156.s004.docx]

**S1 file: Materials and Methods**

**Phage display peptide library screening**

To verify the purity and concentration of FvTox1, we ran a FvTox1 sample on the SDS-PAGE, and then stained with Bio-Rad silver stain (Bio-Rad Laboratories, Inc., Hercules, CA). The Bio-rad Bradford protein quantitation reagent was used to determine the FvTox1 concentration, and the toxin was diluted to 30 ng/μl in 0.1 M NaHCO_3_, pH 8.6. 1.5 ml of FvTox1 solution in 0.1 M NaHCO_3_, pH 8.6 was added in each well of the 12-well Corning cell culture plates. Plates were incubated overnight at 4°C with gentle agitation. The coating FvTox1 solution was replaced with blocking buffer (5 mg/ml BSA in 0.1 M NaHCO_3_) and incubated at 4°C for at least 1 h with shaking. The blocking buffer was poured off and the plate was washed for six times with TBST buffer (TBS containing 0.1% Tween 20). From each of the three phage display peptide libraries, 10 μl was diluted in 1 ml TBST buffer and placed in the well of the culture plate and shaken gently for 60 min at room temperature. The unbound phage particles were removed by pouring off the phage solution and then washed the plate ten times with TBST buffer. The phage particles that were attached to the plate, presumably through interaction with FvTox1, were eluted with 1 ml elution buffer (0.2 M glycine-HCl, pH 2.2). To this suspension, BSA (1 mg/ml) was added and shaken gently for 20 min. The elution buffer was neutralized with 150 μl of 1 M Tris-HCl, pH 9.1. The resultant eluate was used to generate a serial dilution (10^-1^ to 10^-3^) of plaque forming units (pfu) with sterile water. The serial dilutions were used to infect 200 μl of *E. coli* ER2738 cells (1:100 dilution of overnight culture) to titer the phages in the eluates. To amplify phage particles, *E. coli* ER2738 cells were infected with individual phage particles of the remaining eluate and incubated for 5 h with vigorous shaking at 37°C. At the end of a 5 h incubation period, the cultures were spun at 1,2000 g for 15 min at 4°C. The pellets were re-suspended in 1 ml of TBS buffer by vigorous shaking. To the phage suspension, 1/6th volume of 20% PEG in 2.5 M NaCl was added and mixed by with vortexing. The mixture was incubated on ice for 60 min and the pellet was collected by centrifugation at 14,000 rpm for 10 min at 4°C. The pellet was resuspended in 200 μl of TBS buffer. The phage solutions were titered by infecting the *E. coli* ER2738 cells with a serial dilution (10^-6^ through 10^-9^) of the phage suspension. The entire screening procedure was repeated two more times to enrich the FvTox1-interacting M13 phage particles. At the end of the third round panning, the eluate without amplification was used to conduct western blot analyses for identifying positive phage clones that bind to FvTox1.

**Western blotting**

After three rounds of panning, the final eluate was diluted for plating at a concentration of <300 pfu in individual Petri plates (9 cm diameter) carrying X-gal/IPTG amended LB agar medium. To lift plaques onto nitrocellulose membrane, the nitrocellulose membranes were first immersed in distilled water for one min and then air-dried for 10 min at room condition. An activated membrane was then placed on each plate with uniform contact with the plaques. The plates with membrane were incubated at 37°C for 2 h, and membrane was marked with a needle before lifting the membrane from the plate. The membranes were then washed three times with TBS buffer, each wash for 10 min. The membranes were blocked with 3% BSA at room temperature for 1 h, then washed for 3 times with TBS buffer with each wash for 5 min. Twenty ml of FvTox1 solution (1 ng FvTox1/μl) in TBS buffer was added to each membrane after the final wash, and incubated overnight at 4°C. The membranes were washed for three times, each for 5 min with TBS buffer. The anti-His antibody was then added to the membrane and incubated at room temperature for 1 h with gentle shaking. After washing three times with PBS buffer, mouse secondary antibody conjugated with alkaline phosphatase (AP) was applied to membranes and incubated for 1 h with gentle shaking. The membranes were then washed three times with PBS buffer, each wash for 5 min. The AP conjugate substrate (Bio-Rad, Laboratories, Inc. Hercules, CA) was added to the membrane after the final wash and incubated for 1 h to overnight for color development.

Each positive phage clone was mixed with 40 μl sterile water to prepare phage clone stocks and used to determine their interactions with FvTox1 by conducting western blot analyses as follows. First, 3 μl drops of 100 ng/μl FvTox1 solution was placed on a nitrocellulose membrane and air-dried for 5 min. After blocking with BSA, membrane pieces carrying FvTox1 were incubated in individual phage particles (1 ml containing 10^14^ pfu) for overnight at 4°C. Anti-M13 pIII monoclonal antibody (New England Lab, Woburn, MA) and AP conjugated secondary antibody were used to determine the interaction of the M13 phage particles to FvTox1. Finally the plasmid DNA from each selected phage particle was extracted for sequencing at the ISU DNA facility.

**Yeast two hybrid and β-galactosidase activity assay**

Synthetic FvTox1-interacting peptide genes were PCR amplified and cloned into the pB42D vector. FvTox1 was cloned into pLexA vector. FvTox1-pLexA plasmid was individually co-transformed with each of the 18 synthetic FvTox1-interacting peptide genes into EGY48 [pSH18-34] cells which carry two reporters, LacZ and LEU2. The transformed cells were plated on minimal agar plates (SD/-His/-Trp/-Ura) to select colonies containing both plasmids. To test the activation of both reporter genes (LacZ and LEU2), 5 clones from each transformation were selected to individually inoculate 3 ml of SD/Glucose/-His/-Trp/-Ura liquid medium and grow for overnight. The next day, the cultures were harvested and resuspended in distilled water and diluted to an OD 600 of 0.8, and 2 μl of the diluted culture was plated on an LB agar amended with SD/Gal/Raf/-His/-Trp/-Ura/-leu and X-gal. Plates were incubated at 30°C for 3 days. In parallel, two FvTox1-interacting soybean proteins identified earlier in a separate study (N. Pudake and M.K. Bhattacharyya, unpublished) were used as positive controls, and a protein that does not interact with FvTox1 as a negative control. The experiment was repeated twice. For β -galactosidase activity assay, yeast clones were inoculated in 3 ml SD/Glucose/-His/-Trp/-Ura liquid medium and incubated for overnight at 30°C. Overnight grown cultures were vortexed for 0.5 min to break the cell clumps. One ml of the overnight culture was then mixed with 4 ml SD/Gal/Raf induction liquid medium and incubated at 30°C for 3-5 h with shaking until the OD 600 of the cells was a little over 0.5. One and a half ml of culture is dispensed into an 1.5 ml tube and centrifuged at 14,000 rpm for 30 s to pellet the cells. The supernatant is removed and 1.0 ml of Buffer 1 (100 ml: 2.38 g HEPES, 0.9 g NaCl, 0.065 g L-Aspartate, 1 g BSA, 50 μl Tween 20) was added to resuspend the cells thoroughly. The cells were pelleted once again, finally resupsended in 300 μl of Buffer 1 and 0.1 ml of the cell suspension was transferred to a fresh tube. The tubes were placed in liquid nitrogen for 1 min, and the frozen tubes were then transferred to a 42°C water bath for 1 min to thaw the cells. The freeze/thaw cycle was repeated two more times and 0.7 ml of Buffer 2 (27.1 mg CPRG in 20 ml of Buffer 1) was then added and mix thoroughly. The time of adding Buffer 2 was recorded. When the color of the samples was turning from yellow/grey to red, 0.5 ml of 3.0 mM ZnCl_2_ solution was added to each tube. The samples were centrifuged at 14,000 rpm for 1 min to pellet cell debris. The supernatant is then transferred to a fresh Eppendorf tube. Using the Buffer 2 as the blank, the OD of the supernatant at 578 nm was recorded. β-galactosidase activity was calculated as follows: β-galactosidase activity = 1,000*OD at 578 nm/(t*V*OD600); where, t = elapsed time (min) of incubation; V = 0.1*concentration factor; OD 600 = absorbance of 1 ml culture at 600 nm.

**Pull down assay**

To prepare GST-tagged FvTox1, FvTox1 was cloned into the plasmid vector, pET41 in *E. coli* DH10B. The resultant plasmid construct was sequenced at the ISU DNA facility for confirmation, and then transformed into the *E. coli* BL21 (DE3) pLysS cells. The IPTG induced cells were sonicated and centrifuged to collect supernatant. The supernatant was incubated with glutathione resin at 4°C for 2 h. The resin was then washed three times with PBS buffer and resuspended in 0.5 ml PBS buffer to prepare the immobilized GST tagged FvTox1. SDS PAGE was run to determine the concentration of purified protein. Immobilized GST-tagged FvTox1 (2 μg) was mixed to each purified His-tagged synthetic peptide (5 μg) (Table 3; Fig. 2). The mixtures were incubated at 4°C for 60 min with agitation, then spin to collect GST resin. The resin was washed five times with 1 ml PBST (PBS buffer with 0.05% Tween-20), and then resuspended in 30 μl PBS buffer to conduct SDS PAGE. Each pull down sample of 15 μl and individual synthetic peptides of 10 μl were loaded for running 15% SDS PAGE. Anti-His primary antibody and HRP conjugated anti-mouse secondary antibody were used to conduct western blot analyses.

**Stem cutting assay**

The stem cutting assay was conducted according to Brar et al. [7]. For preparing cell-free culture filtrates, 40 mycelial plugs from 14 day-old *F. virguliforme* culture on solid Bilay medium (0.1% KH_2_PO_4_ [w/v], 0.1% KNO_3_ [w/v], 0.05% MgSO_4_[w/v], 0.05% KCl [w/v], 0.02% starch [w/v], 0.02% glucose [w/v], and 0.02% sucrose [w/v]) plates were used to inoculate 200 ml Bilay liquid medium and incubated under dark at room temperature for 12 days. The culture was then filtered sequentially through Mira cloth (EMD Millipore, Billerica, MA) and then 0.2 μm sterile syringe filter (Cornning Inc., NY). The final filtrate was stored at 4°C prior to use.

One ml cell-free *F. virguliforme* culture filtrate was mixed individually to 5 μl each of the four synthetic peptides (10 μg/μl) with no His tags (Table 4), and mixtures were incubated overnight at 4°C. The mixture was then added in a 50 ml tube containing 40 ml water. A single 3-week old Williams 82 seedling, grown in a Conviron under the condition of 16 h light and 8 h dark period, was cut below the cotyledons and placed into each of these tubes containing diluted mixture of cell-free *F. virguliforme* culture filtrates and peptides. Cut soybean seedlings were also placed in 40 ml water to serve as a negative control, and in 40 ml water mixed with 1 ml cell-free *F. virguliforme* culture filtrates to serve as a positive control. Seedlings were placed back to the Conviron for symptom development. Symptoms were started to develop 3-4 days following feeding with diluted cell-free *F. virguliforme* culture filtrates. The disease symptoms were scored 7 and 8 days following feeding of cell-free *F. virguliforme* culture filtrates with or without the FvTox1-interacting peptides. The scoring scheme was similar to the one reported earlier [9]: 1, no symptoms; 2, <10% chlorosis; 3, 10 to 20% chlorosis; 4, 20-50% chlorosis; 5, 50 to 80% chlorosis and necrosis; 6, entire leaf was chlorotic or necrotic. There were five replications in each treatment and the experiment was conducted three times.

For determination of chlorophyll contents, soybean leaf disks of 2 cm diameter were cut and placed individually in Eppendorf tubes. In each tube, 1 ml of 80% acetone was added. Subsequently, the tubes were incubated at room temperature in the dark for 5 days. Absorbance of the acetone solution containing chlorophyll was measured at 645 and 663 nm. The amount of chlorophyll contents were calculated according to the method described earlier [1].

**References**

1. Arnon DI. Copper enzymes in isolated chloroplasts. Polyphenoloxidase in *Beta vulgaris*. Plant Physiol. 1949;24: 1–15.
