# Supplementary material for: Identification of Fusarium virguliforme FvTox1-Interacting Synthetic Peptides for Enhancing Foliar Sudden Death Syndrome Resistance in Soybean
Source: PLoS One. 2015 Dec 28;10(12):e0145156. doi: 10.1371/journal.pone.0145156 (PMC4692527; doi:10.1371/journal.pone.0145156)
Supplement: S1 Table — (DOCX) [file pone.0145156.s005.docx]

**S1 Table. Primers used in this study.**

| Name | Enzyme | Sequence (5’ to 3’) |
| --- | --- | --- |
| PB42-Ncys-F | *Sal*l | CGCGTCGACATGggt CGGGATCTGTACGAC |
| PB42-Ncys-R | *Sal*l | CGCGTCGACGCAGCCGGATCAAGCTTC |
| PB42-cys-F | *Sal*l | CGCGTCGACATGTGC CGGGATCTGTACGAC |
| PB42-cys-R | *Sal*l | CGCGTCGACGCAGCCGGATCAAGCTTC |
| phage1 |  | GCCCCCTCCGCCCAGGCGGTATTCGTAAATGGTCTCGGGAAGATAGGAACTTCCACCTCC |
| Phage2 |  | GCCCCCTCCGCCAACCTCGCGATCGTGGTATCTGGTCTTATTCTCCACACTTCCACCTCC |
| Phage3 |  | GCCCCCTCCGCCAACAGACCTGGCGTAATTGTGCCAAGCACCCTCGTGACTTCCACCTCC |
| Phage4 |  | GCCCCCTCCGCCATCTGCAACTCTCCCGTTGCTACTTCCACCTCC |
| Phage-*Bam*HI-F | *Bam*HI | CGCGGATCCGGTGGGGGCGGATCA |
| Phage-EcoRl-R | *Eco*Rl | CCGGAATTCTTATGAGCCCCCTCCGCC |
| Phage-Sacl-F | *Sac*l | CGCGAGCTCGGTGGGGGCGGATCA |
| Phage-Sacl-R | *Sac*l | CGCGAGCTCTGAGCCCCCTCCGCC |
| Phage-Xhol-F | *Xho*l | CCGCTCGAGGGTGGGGGCGGATCA |
| Phage- Xhol-R | *Xho*l | CCGCTCGAGTGAGCCCCCTCCGCC |
| Phage-Kpnl-F | *Kpn*l | CGGGGTACCGGTGGGGGCGGATCA |
| FvTox1-F | *Bam*HI | AACGGGATCCATGGGCAGCGCAGCCAGATAC |
| FvTox1-R | *Hind*III | GGAGATAAGCTTCTG CTGTGGGTTGCGCACACA |
