# Supplementary material for: Identification of Fusarium virguliforme FvTox1-Interacting Synthetic Peptides for Enhancing Foliar Sudden Death Syndrome Resistance in Soybean
Source: PLoS One. 2015 Dec 28;10(12):e0145156. doi: 10.1371/journal.pone.0145156 (PMC4692527; doi:10.1371/journal.pone.0145156)
Supplement: S2 Table — (DOCX) [file pone.0145156.s006.docx]

**S2 Table. Nucleotide sequences of all nine peptides.**

| **Gene** | **Nucleic acid sequence** |
| --- | --- |
| **1** | GGTAGTACTGCAATTGGTATGAAAGAAACCGCTGCTGCTAAATTCGAACGCCAGCACATGGACAGCCCAGATCTGGGTACCGGTGGTGGCTCCGGTGATGACGACGACAAGAGTCCCATGGGATATCGGGGATCCGGTGGGGGCGGATCAGGCGGTGGTGGTAGTGGAGGAGGTGGAAGTTCCTATCTTCCCGAGACCATTTACGAATACCGCCTGGGCGGAGGGGGCTCACTCGAGCACCACCACCACCACCACCACCACTAA |
| **2** | GGTAGTACTGCAATTGGTATGAAAGAAACCGCTGCTGCTAAATTCGAACGCCAGCACATGGACAGCCCAGATCTGGGTACCGGTGGTGGCTCCGGTGATGACGACGACAAGAGTCCCATGGGATATCGGGGATCCGGTGGGGGCGGATCAGGCGGTGGTGGTAGTGGAGGAGGTGGAAGTGTGGAGAATAAGACCAGATACCACGATCGCGAGGTTGGCGGAGGGGGCTCACTCGAGCACCACCACCACCACCACCACCACTAA |
| **3** | GGTAGTACTGCAATTGGTATGAAAGAAACCGCTGCTGCTAAATTCGAACGCCAGCACATGGACAGCCCAGATCTGGGTACCGGTGGTGGCTCCGGTGATGACGACGACAAGAGTCCCATGGGATATCGGGGATCCGGTGGGGGCGGATCAGGCGGTGGTGGTAGTGGAGGAGGTGGAAGTCACGAGGGTGCTTGGCACAATTACGCCAGGTCTGTTGGCGGAGGGGGCTCACTCGAGCACCACCACCACCACCACCACCACTAA |
| **4** | GGTAGTACTGCAATTGGTATGAAAGAAACCGCTGCTGCTAAATTCGAACGCCAGCACATGGACAGCCCAGATCTGGGTACCGGTGGTGGCTCCGGTGATGACGACGACAAGAGTCCCATGGGATATCGGGGATCCGGTGGGGGCGGATCAGGCGGTGGTGGTAGTGGAGGAGGTGGAAGTAGCAACGGGAGAGTTGCAGATGGCGGAGGGGGCTCACTCGAGCACCACCACCACCACCACCACCACTAA |
| **5** | ATGCGGGGTTCTCATCATCATCATCATCATGGTATGGCTAGCATGACTGGTGGACAGCAAATGGGTCGGGATCTGTACGACGATGACGATAAGGATCGATGGGAGGATCCGGTGGGGGCGGATCAGGCGGTGGTGGTAGTGGAGGAGGTGGAAGTTCCTATCTTCCCGAGACCATTTACGAATACCGCCTGGGCGGAGGGGGCTCAGAGCTCGGTGGGGGCGGATCAGGCGGTGGTGGTAGTGGAGGAGGTGGAAGTGTGGAGAATAAGACCAGATACCACGATCGCGAGGTTGGCGGAGGGGGCTCA |
| **6** | ATGCGGGGTTCTCATCATCATCATCATCATGGTATGGCTAGCATGACTGGTGGACAGCAAATGGGTCGGGATCTGTACGACGATGACGATAAGGATCGATGGGAGGATCCGGTGGGGGCGGATCAGGCGGTGGTGGTAGTGGAGGAGGTGGAAGTCACGAGGGTGCTTGGCACAATTACGCCAGGTCTGTTGGCGGAGGGGGCTCAGAGCTCGGTGGGGGCGGATCAGGCGGTGGTGGTAGTGGAGGAGGTGGAAGTAGCAACGGGAGAGTTGCAGATGGCGGAGGGGGCTCA |
| **7** | ATGCGGGGTTCTCATCATCATCATCATCATGGTATGGCTAGCATGACTGGTGGACAGCAAATGGGTCGGGATCTGTACGACGATGACGATAAGGATCGATGGGAGGATCCGGTGGGGGCGGATCAGGCGGTGGTGGTAGTGGAGGAGGTGGAAGTTCCTATCTTCCCGAGACCATTTACGAATACCGCCTGGGCGGAGGGGGCTCAGAGCTCGGTGGGGGCGGATCAGGCGGTGGTGGTAGTGGAGGAGGTGGAAGTGTGGAGAATAAGACCAGATACCACGATCGCGAGGTTGGCGGAGGGGGCTCACTCGAGGGTGGGGGCGGATCAGGCGGTGGTGGTAGTGGAGGAGGTGGAAGTCACGAGGGTGCTTGGCACAATTACGCCAGGTCTGTTGGCGGAGGGGGCTCA |
| **8** | ATGCGGGGTTCTCATCATCATCATCATCATGGTATGGCTAGCATGACTGGTGGACAGCAAATGGGTCGGGATCTGTACGACGATGACGATAAGGATCGATGGGAGGATCCGGTGGGGGCGGATCAGGCGGTGGTGGTAGTGGAGGAGGTGGAAGTTCCTATCTTCCCGAGACCATTTACGAATACCGCCTGGGCGGAGGGGGCTCAGAGCTCGGTGGGGGCGGATCAGGCGGTGGTGGTAGTGGAGGAGGTGGAAGTGTGGAGAATAAGACCAGATACCACGATCGCGAGGTTGGCGGAGGGGGCTCACTCGAGGGTGGGGGCGGATCAGGCGGTGGTGGTAGTGGAGGAGGTGGAAGTAGCAACGGGAGAGTTGCAGATGGCGGAGGGGGCTCA |
| **9** | ATGCGGGGTTCTCATCATCATCATCATCATGGTATGGCTAGCATGACTGGTGGACAGCAAATGGGTCGGGATCTGTACGACGATGACGATAAGGATCGATGGGAGGATCCGGTGGGGGCGGATCAGGCGGTGGTGGTAGTGGAGGAGGTGGAAGTTCCTATCTTCCCGAGACCATTTACGAATACCGCCTGGGCGGAGGGGGCTCAGAGCTCGGTGGGGGCGGATCAGGCGGTGGTGGTAGTGGAGGAGGTGGAAGTGTGGAGAATAAGACCAGATACCACGATCGCGAGGTTGGCGGAGGGGGCTCA |
