# Supplementary material for: Identification of Fusarium virguliforme FvTox1-Interacting Synthetic Peptides for Enhancing Foliar Sudden Death Syndrome Resistance in Soybean
Source: PLoS One. 2015 Dec 28;10(12):e0145156. doi: 10.1371/journal.pone.0145156 (PMC4692527; doi:10.1371/journal.pone.0145156)
Supplement: S3 Table — (DOCX) [file pone.0145156.s007.docx]

**S3 Table.** **Fusion peptides expressed in *E. coli* for pull down assay.**

| Protein | Sequence | Length/Mw/PI |
| --- | --- | --- |
| 1 | GSTAIGMKETAAAKFERQHMDSPDLGTGGGSGDDDDKSPMGYRGSGGGGSGGGGSGGGGSSYLPETIYEYRLGGGGSLEHHHHHHHH- | 87/8.72/5.93 |
| 2 | GSTAIGMKETAAAKFERQHMDSPDLGTGGGSGDDDDKSPMGYRGSGGGGSGGGGSGGGGSVENKTRYHDREVGGGGSLEHHHHHHHH- | 87/8.72/6.17 |
| 3 | GSTAIGMKETAAAKFERQHMDSPDLGTGGGSGDDDDKSPMGYRGSGGGGSGGGGSGGGGSHEGAWHNYARSVGGGGSLEHHHHHHHH- | 87/8.6/6.23 |
| 4 | GSTAIGMKETAAAKFERQHMDSPDLGTGGGSGDDDDKSPMGYRGSGGGGSGGGGSGGGGSSNGRVADGGGGSLEHHHHHHHH- | 82/7.89/6.10 |
| 5 | MRGSHHHHHHGMASMTGGQQMGRDLYDDDDKDRWGSGGGGSGGGGSGGGGSSYLPETIYEYRLGGGGSELGGGGSGGGGSGGGGSVENKTRYHDREVGGGGS- | 102/9.97/5.90 |
| 6 | MRGSHHHHHHGMASMTGGQQMGRDLYDDDDKDRWGSGGGGSGGGGSGGGGSHEGAWHNYARSVGGGGSELGGGGSGGGGSGGGGSSNGRVADGGGGS- | 97/9.02/6.19 |
| 7 | MRGSHHHHHHGMASMTGGQQMGRDLYDDDDKDRWGSGGGGSGGGGSGGGGSSYLPETIYEYRLGGGGSELGGGGSGGGGSGGGGSVENKTRYHDREVGGGGSLEGGGGSGGGGSGGGGSHEGAWHNYARSVGGGGS- | 136/12.88/5.93 |
| 8 | MRGSHHHHHHGMASMTGGQQMGRDLYDDDDKDRWGSGGGGSGGGGSGGGGSSYLPETIYEYRLGGGGSELGGGGSGGGGSGGGGSVENKTRYHDREVGGGGSLEGGGGSGGGGSGGGGSSNGRVADGGGGS- | 131/12.17/5.71 |
| 9 | MRGSHHHHHHGMASMTGGQQMGRDLYDDDDKDRWGSGGGGSGGGGSGGGGSSYLPETIYEYRLGGGGSELGGGGSGGGGSGGGGSVENKTRYHDREVGGGGSLEGGGGSGGGGSGGGGSHEGAWHNYARSVGGGGSGTGGGGSGGGGSGGGGSSNGRVADGGGGS- | 165/15/5.93 |
