# Supplementary material for: Identification of Fusarium virguliforme FvTox1-Interacting Synthetic Peptides for Enhancing Foliar Sudden Death Syndrome Resistance in Soybean
Source: PLoS One. 2015 Dec 28;10(12):e0145156. doi: 10.1371/journal.pone.0145156 (PMC4692527; doi:10.1371/journal.pone.0145156)
Supplement: S4 Table — (DOCX) [file pone.0145156.s008.docx]

**S4 Table. Plasmid constructs included in this study.**

| Plasmid name | Plasmid name |
| --- | --- |
| pET41-peptide1 | pB42AD-Nocys-peptide1 |
| pET41-peptide2 | pB42AD- Nocys-peptide2 |
| pET41-peptide3 | pB42AD- Nocys-peptide3 |
| pET41-peptide4 | pB42AD- Nocys-peptide4 |
| pRSET-peptide1+2 | pB42AD- Nocys-peptide1+2 |
| pRSET-peptide3+4 | pB42AD- Nocys-peptide3+4 |
| pRSET-peptide1+2+3 | pB42AD- Nocys-peptide1+2+3 |
| pRSET-peptide1+2+4 | pB42AD- Nocys-peptide1+2+4 |
| pRSET-peptide1+2+3+4 | pB42AD- Nocys-peptide1+2+3+4 |
| pRSET-FvTox1 | pB42AD-cys-peptide1 |
| pET28-FvTox1 | pB42AD- cys-peptide2 |
| pET41-FvTox1 | pB42AD- cys-peptide3 |
|  | pB42AD- cys-peptide4 |
|  | pB42AD- cys-peptide1+2 |
|  | pB42AD- cys-peptide3+4 |
|  | pB42AD- cys-peptide1+2+3 |
|  | pB42AD- cys-peptide1+2+4 |
|  | pB42AD- cys-peptide1+2+3+4 |
